# Self-DNA Exposure Induces Developmental Defects and Germline DNA Damage Response in *Caenorhabditis elegans*

**DOI:** 10.3390/biology11020262

**Published:** 2022-02-08

**Authors:** Marcello Germoglio, Adele Adamo, Guido Incerti, Fabrizio Cartenì, Silvia Gigliotti, Aurora Storlazzi, Stefano Mazzoleni

**Affiliations:** 1 Institute of Biosciences and BioResources, National Research Council, Via Pietro Castellino 111, 80131 Napoli, Italy; m.germo87@gmail.com (M.G.); adele.adamo@ibbr.cnr.it (A.A.); silvia.gigliotti@ibbr.cnr.it (S.G.); 2 Department of Agri-Food, Animal and Environmental Sciences (DI4A), University of Udine, 33100 Udine, Italy; guido.incerti@uniud.it; 3 Department of Agricultural Sciences, Università degli Studi di Napoli Federico II, 80055 Portici, Italy; fabrizio.carteni@unina.it

**Keywords:** *Caenorhabditis elegans*, self-DNA, germline, apoptosis, nematode

## Abstract

**Simple Summary:**

All organisms, from bacteria to mammals, activate responses protecting themselves from dangers represented by outsider invaders and damages. Inappropriately localised self-DNA is one of the molecular clues detected as a danger and triggering defence reactions that may lead to chronic activation of inflammatory conditions. In this study, we investigate if dietary delivered self-DNA is detrimental in a simple metazoan model organism, the nematode Caenorhabditis elegans. Adverse effects were observed in the progenies of worms exposed to self-DNA integrated into their bacterial diet. The presence of self-DNA in the food significantly decreased egg deposition, induced high embryo death, and negatively affected larval development. The findings, on the one side, raise interesting questions on the basic molecular mechanisms involved in response to extracellular self-DNA. On the other side, the observed phenomenon suggests possible applications for the biocontrol of parasitic nematodes by appropriate delivery of their self-DNA in their growing environment.

**Abstract:**

All organisms, from bacteria to mammals, sense and respond to foreign nucleic acids to fight infections in order to survive and preserve genome integrity across generations. The innate immune system is an evolutionarily conserved defence strategy. Complex organisms have developed various cellular processes to respond to and recognise not only infections, i.e., pathogen-associated molecular patterns (PAMPs), but also to sense injury and tissue dysfunctions, i.e., damage-associated molecular patterns (DAMPs). Mis-localized self-DNA can be sensed as DAMP by specific DNA-sensing pathways, and self-DNA chronic exposure can be detrimental to the organisms. Here, we investigate the effects of dietary delivered self-DNA in the nematode *Caenorhabditis elegans*. The hermaphrodite worms were fed on *Escherichia coli* genomic libraries: a *C. elegans* library (self) and a legume (*Medicago truncatula*) library (non-self). We show that the self-library diet affects embryogenesis, larval development and gametogenesis. DNA damage and activation of p53/CEP-1-dependent apoptosis occur in gonadal germ cells. Studies of self-DNA exposure in this model organism were not pursued up to now. The genetic tractability of *C. elegans* will help to identify the basic molecular pathways involved in such mechanisms. The specificity of the adverse effects associated with a self-DNA enriched diet suggests applications in biological pest control approaches.

## 1. Introduction

Foreign nucleic acids are targets of multiple and sophisticated mechanisms aimed at maintaining genome integrity in all types of invaded cells. As part of the immune response, specific molecular pathways are activated to sense both foreign DNA and damaged or aberrantly localised self-DNA inside the cell [1]. To avoid unwanted responses to regular physiological processes, recognition of endogenous nucleic acids is actively regulated by the cells and alteration of such mechanisms is associated with various diseases [2].

In plants, it was reported that extracellular, fragmented, self-DNA accumulating in litter during the decomposition process has a concentration-dependent inhibitory effect on seed germination and roots elongation [3]. It was further assessed that growth inhibition due to the presence of self-DNA in the growth medium was a general biological response [4]. Growth of organisms belonging to different taxonomic groups, including a bacterium, a fungus, an alga, an amphibian, a protozoan, a dipteran and a plant, was unaffected in the presence of heterologous DNA and negatively affected by increasing concentrations of self-DNA. The effect of self-DNA in plants was further analysed and found to activate defence-related responses acting as a damage-associated molecular pattern (DAMP) [5], whereas a very recent study revealed a much more complex response at the transcriptional level leading in time to a cascade of events specific to extracellular self-DNA [6]. In multicellular organisms, DAMPs are associated with infections or cellular damage. Once detected, DAMPS elicit immune responses against the invading organisms and promote damage repair [7].

Herein, we extend the study of self-DNA inhibition to a model nematode, *Caenorhabditis elegans*.

*C. elegans* is an excellent model to address questions concerning DAMPs related immune pathways, as it does not seem to detect pathogens per se but uses effector-triggered and DAMP-triggered immunity to respond to the consequences of infections [8]. In this organism, infection and damage-activated pathways result interwoven with stress-response pathways. For example, significant overlap in the transcriptional responses to osmotic stress and certain infections was reported [9]. Genotoxic stresses that activate the DNA damage response (DDR) are linked to cellular immune response [10]. It was shown in *C. elegans* that physiologically induced double-strand breaks (DSBs), mediated by the SPO-11 topoisomerase during meiosis, confer systemic stress resistance [11]. Cells devoted to gametogenesis are the only proliferative cells in adult *C. elegans*, and at a specific stage in the gonads of the hermaphrodite before oocytes maturation, many meiotic cells undergo physiologically regulated apoptosis as part of the regular programme. Interestingly a specific increase in apoptosis in the germline is one of the outcomes of intestinal infection with *Salmonella typhimirium*, and worms mutated in genes required to accomplish this apoptotic programme exhibit increased susceptibility to the same bacteria [12].

In this study, the following hypotheses are investigated: (i) Is the inhibitory effect of self-DNA observed also in a nematode? (ii) Is a feeding diet based on a genomic library in a bacterial vector an effective administration option for self-DNA delivery to a target organism? (iii) Which adverse effects can be observed during the different developmental stages? (iv) Does the exposure to extracellular self-DNA induce DNA damage?

## 2. Materials and Methods

### 2.1. Caenorhabditis elegans and Escherichia coli Strains

The following *C. elegans* strains were provided by the *Caenorhabditis* Genetics Centre: wild-type Bristol (N2); WM27 *rde-1*(ne219) V; CB1392 *nuc-1*(e1392) X; AV106 *spo-11*(ok79) IV/nT1[unc-?(n754) let-?] IV;V; VC172 cep-1(gk138) III.

*E. coli* OP50 strain was used as standard *C. elegans* food source. *E. coli* EPI300T1^R^ is the bacterial strain hosting the fosmid libraries used in this study (“Source BioScience”).

### 2.2. Fosmid Libraries

The *C. elegans* and *Medicago truncatula* fosmid libraries were both constructed in *E. coli* EPI300-T1^R^ in the fosmid Copy Control vector pCC1FOS (chloramphenicol resistance, fosmid copy number inducible by arabinose).

The *C. elegans* library was purchased from “Source BioScience”. It consists of 15,744 indexed bacterial clones (average insert size of 43.3 kb) distributed on 41 multiwell plates.

The *Medicago truncatula* library was purchased from “INRA-CNRGV” and consists of 68,352 clones (average insert size of 40 kb). It was purchased as a pooled library distributed on two multiwell plates in 178 pools.

### 2.3. Culture Conditions

*C. elegans* strains were maintained at 20 °C on nematode growth medium (NGM) agar plates seeded with *E. coli* OP50 lawn [13]. The fosmid libraries were stored in glycerol (30% vol/vol) at −80 °C as pooled clones.

Pools of the *C. elegans* library were constructed growing, on large Petri dishes containing chloramphenicol solid agar LB, all the clones of each multiwell plate as isolated spots. All the spots were mixed and collected in 12 mL of LB with the help of a spreader. Finally, all bacteria were mixed and resuspended in glycerol (30% vol/vol), and aliquots were frozen at −80 °C. These bacteria aliquots were inoculated in LB supplemented with chloramphenicol (12.5 μg/mL) and L-arabinose (0.01%, L-arabinose omitted when specified in the text) and used as a food source (self) on NGM plates. Each aliquot of the pools of the *C. elegans* library was frozen back at −80 °C no more than two times.

The two plates containing 178 pools of the *Medicago truncatula* library were also replicated on large Petri dishes, bacteria spots were collected, and aliquots of each mixed pool obtained were frozen separately. Each pool was separately grown in LB supplemented with chloramphenicol and L-arabinose and finally mixed and used as a food source (non-self) on NGM plates.

### 2.4. Determination of Average Fosmid Copy Number

Total DNA was purified from pooled *C. elegans* and *M. truncatula* fosmid libraries, grown 4, 8 and 16 h in LB supplemented with chloramphenicol and L-arabinose and 16 h in LB supplemented with chloramphenicol, using the Purification Kit Wizard Genomic DNA (Promega) according to the manufacturer’s instructions.

Relative fosmid copy number was determined by real-time quantitative PCR (qPCR) [14]. qPCR reaction samples (20 μL) consisted of 12.5 μL Power SYBR Green PCR Master Mix (Applied Biosystem), 15 pmol of each primer and 2 ng DNA template. The *cat* gene on the fosmid vector and the *dxs* gene on the *E. coli* chromosome were amplified using CmR-qPCR-F (5′- GGGAAATAGGCCAGGTTTTC-3′) and CmR-qPCR-R (5′- TCCATGAGCAAACTGAAACG-3′) primers and Upper EPI300 (5′-GTCATTATGACCCCG-3′) and Lower EPI300 (5′-GGTAGTTTTTCCAGC-3′) primers, respectively. An initial denaturation step at 94 °C for 2 min was followed by 40 amplification cycles (30 s at 94 °C, 30 s at 55 °C and 1 min at 72 °C). qPCR results were recorded as fosmid copy number fold changes after normalising for the E. *coli* genomic *dxs* gene and computed using the comparative ΔΔCT method (2^−ΔΔCT^) [15]. Standard error was calculated from 3 technical replicates.

### 2.5. Phenotype Screening in C. elegans

Young adult hermaphrodite worms, grown at 20 °C on NGM agar plates spread with *E. coli* OP50 as a food source, were individually transferred to fresh NGM plates, with or without ([A−]) L-arabinose, seeded with large lawns of the pooled clones of either library and incubated at 20 °C. Each worm was transferred twice a day onto a fresh identical plate until all fertilised eggs were laid (3 days).

To measure embryonic lethality, eggs were scored 24 h after laying, and the ratio of unhatched eggs to the total laid eggs was calculated [16]. Brood size was defined as the number of eggs laid by each worm in a 3-day time interval. Larval arrests/delays and aberrant phenotypes were monitored up to 96 h after egg laying.

First-generation (F_1_) healthy (by visual inspection) young adult worms were picked from the third-day egg collections, individually cloned on fresh NGM plates containing L-arabinose and seeded with self or non-self libraries and incubated at 20 °C to score brood size, embryonic lethality and larval/adult defective phenotypes.

### 2.6. Time Course of Larval Development (from Hatching to Adulthood)

Young adult hermaphrodite worms were individually transferred to fresh NGM plates, with L-arabinose, seeded with the pooled clones of either library and allowed to lay eggs for 12 h at 20 °C. The number of individuals that reached the adult stage in the time interval of 60–96 h after egg laying was scored. There were 10 self- and 9 non-self-scored plates for each generation.

### 2.7. Immunostaining in C. elegans Germline

Gonads of F_1_ adults fed on self and non-self libraries were dissected in M9 Buffer (0.3% H_2_PO_4_, 0.6% Na_2_HPO_4_, 0.5% NaCl and 1 mM MgSO_4_) on Polylysine glass slides. The specimens were freeze-cracked in liquid nitrogen, sequentially immersed, at −20 °C, in methanol, methanol/acetone (1:1) and acetone for 5 min, and washed three times in PBS, for 5 min each. Slides were blocked in 0.3% BSA in PBS for 30 min at 37 °C in a humid chamber. The primary antibody used in this study was rabbit anti-RAD-51 diluted 1:200 in Ab buffer (1% BSA, 0.1% Tween-20, 0.05% sodium azide in PBS). Slides were incubated for 90 min at room temperature followed by three washes in PBS, 5 min each. Secondary antibody was conjugated goat anti-rabbit Texas Red (1:400 in Ab buffer, Invitrogen). Slides were incubated with the antibodies for 60 min in the darkroom at room temperature and washed in PBS + 0.1% Tween-20 for three times, 5 min each. Samples were mounted with Prolong Gold Antifade reagent containing 4′, 6′-diamidino-2- phenylindole hydrochloride (DAPI) (Life Technologies).

The quantitative analysis of RAD-51 foci was performed by dividing the gonad into 5 zones (mitotic zone, transition zone, early pachytene, middle pachytene and late pachytene stage) in accordance with their cytological features, and foci were counted in the early and middle pachytene zones [17]. Five gonads and an average of 100 nuclei for each gonad region were scored for each genotype.

### 2.8. Quantitative Analysis of Germline Apoptosis

Young adult nematodes fed on self and non-self libraries were suspended in M9 Buffer and stained by incubation with 33 µM SYTO-12 (molecular probes) for 1 h and 30 min at room temperature in the dark. The worms were then transferred to seeded plates to allow stained bacteria to be purged from the gut. After 30 min, the animals were mounted on 2% agarose pads in 2 mM levamisole and observed using a Leica DM6 fluorescence microscope. The estimation of apoptotic levels for each genotype was calculated as the average number of apoptotic nuclei per gonadal arm. A total of 90 gonadal arms were observed in three different experiments.

### 2.9. Analysis of DAPI-Stained Bodies in the Germline Nuclei

F_1_ adult nematodes fed on self and non-self libraries were placed in a drop of M9 Buffer on glass slides, permeabilised and fixed with 10 µL of 100% EtOH and directly mounted in 10 µL of DAPI (2 µg/mL) diluted in M9 buffer [18].

### 2.10. Image Collection and Processing

The collection of images was performed using a Leica DM6 fluorescence microscope, equipped with a Hamamatsu camera under the control of Leica LAS X 6000 software. Images were processed and deconvolved using Leica LAS X (version: 3.0.0.15697) software and ImageJ (https://imagej.nih.gov/ij/ accessed on 10 October 2021). Quantitative analyses of RAD-51 foci and DAPI-stained bodies along the gonad were performed on Z-stack images. Optical sections were collected at 0.18 µm and 0.50 µm increments, respectively. The images shown are maximum-intensity projections of Z-stacks.

### 2.11. Statistical Tools

Significant differences among the median percentages of dead embryos and larval arrest/aberrant phenotypes in the F_1_ observed in Self, Self[A+] and Non-self treatments were tested using a non-parametric Kruskal–Wallis one-way analysis of variance, after testing the dependent variables for normality and homoscedasticity assumptions of parametric one-way ANOVA. Pairwise significant differences were tested using a Mann–Whitney U test. Differences among treatments in laid eggs per worm across P_0_–F_3_ generations, as well as in dead embryos and larval arrest/aberrant phenotypes across F_1_–F_4_ generations, were tested using parametric two-way ANOVA models, including first-order and interactive effects of treatment and generation. Pairwise significant differences were tested using Tukey’s post hoc test.

Finally, data on quantification of germline apoptosis in worms fed with indicated bacteria were analysed by one-way ANOVA for significant differences in mean numbers of SYTO-12-labelled nuclei per gonadal arm among the three tested combinations of diet and genotype. Pairwise differences were tested using Tukey’s post hoc test.

## 3. Results

### 3.1. Negative Effects in C. elegans Fed on a self-DNA Bacterial Genomic Library

**Figure 1:**
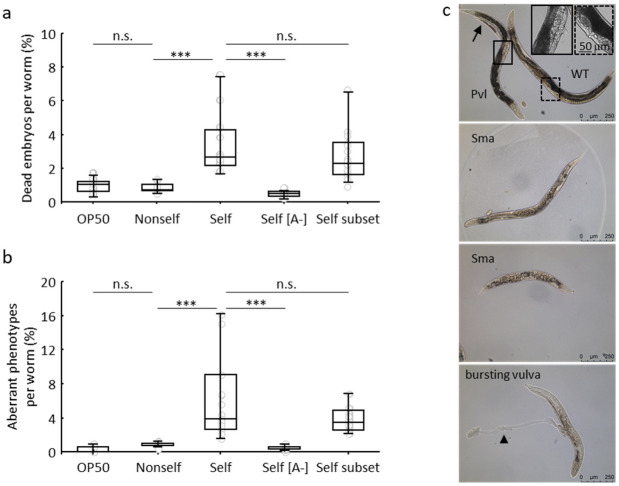
Feeding *C. elegans* with bacteria bearing fosmid libraries. Young adult hermaphrodites, grown on OP50, were transferred to NGM plates (with or without [A-] L-arabinose) seeded with the indicated bacteria. Each parent was transferred to new identically seeded plates twice per day for three days. The number of total eggs laid in this time frame by each parental worm was scored along with unhatched eggs (dead embryos) and larval and adult phenotypes. Number of scored P0 worms: OP50: 8; Non-self: 10; Self: 15; Self[A−]: 10; Self subset: 16. Number of eggs: OP50: 2643; Non-self: 2885; Self: 3872; Self[A−]: 3098; Self subset: 4240. (**a**) Embryonic lethality (unhatched eggs, %). The *y*-axis reports the percentage of dead embryos per worm, and the *x*-axis indicates the diet. (**b**) Aberrant phenotypes (%) observed in the F_1_ progeny. The *y*-axis reports the percentage of aberrant phenotypes (larval arrest/delay and defective adults) per worm, and the *x*-axis indicates the diet. (**c**) Images of wt and defective F_1_ adult worms fed on the self library. Top: high magnification of boxed ventral mid-body region in a wt (WT) and a defective adult worm showing a protruding vulva phenotype (Pvl); the arrow indicates a larval arrest. Central panels: adult worms showing short body length (Sma). Bottom: worm showing bursting vulva and extruded gonad (arrowhead). Scale bars, 250 mm. Grey circles in (**a**,**b**) show % of events per animal. Data in (**a**,**b**) refer to medians, quartiles (boxes) and ranges (whiskers) of the dependent variable calculated for all the worms within each treatment; *** significant between-treatments pairwise differences (Mann–Whitney U test, *p* < 0.001, details in Supplementary Table S1).

*C. elegans* is a bacterivore, and its growth and reproduction have been studied on a variety of bacterial diets [19]. The standard laboratory diet is an *E. coli* OP50 strain, but RNA interference (RNAi) by feeding [20] is based on the *E. coli* HT115 strain engineered to produce double-stranded RNA (dsRNA) [21]. The use of libraries of dsRNA-expressing bacteria as a tool for gene discovery and characterisation inspired our protocol to expose worms to self-DNA. To this purpose, two commercially available genomic fosmid libraries of *C. elegans* and *M. truncatula,* cloned in fosmid copy control vector pCC1FOS and transformed in EPI300-T1^R^ (Epicentre) bacteria, were chosen as nematode diets (self and non-self, respectively). It was previously established that this *E. coli* host (bearing different fosmid libraries) was nontoxic food for *C. elegans* [22]. Indeed, we did not find any apparent difference in worms grown on *E. coli* OP50 or on *M. truncatula* pooled library (Figure 1, Table S1). In pCC1FOS libraries, a conditional increase of fosmid copy number in the bacterial clones is based on the addition of L-arabinose to the growth medium. Real-time quantitative PCR on pooled fosmid library clones, self and non-self, determined that fosmid copy number increased on average 25-fold after sixteen hours of growth in L-arabinose containing LB medium (Figure S1).

To the purpose of our experiment, young adult hermaphrodite worms, developed on a standard *C. elegans* bacterial diet, were individually transferred to nematode growth medium (NGM) plates, with or without ([A-]) L-arabinose, seeded with large lawns of bacteria grown with or without L-arabinose (see Materials and Methods for details).

Self-fertilised eggs laid by single adult worms fed non-self, self and self[A−] libraries, and a subset of seventeen pooled clones of the *C. elegans* library (self subset, see Table S2 for the list of fosmids in the self subset) and the standard OP50 bacteria were collected for three days (see Materials and Methods for details). During this time interval, no phenotypic or behavioural difference was detected in treated adult worms. However, when F_1_ progenies were analysed, the biological impact of the self-DNA enriched diet first became apparent (Figure 1).

Embryonic lethality, measured as the frequency of eggs that did not hatch, was slightly but significantly higher in the offspring of worms fed self compared to non-self and self[A−] libraries (Figure 1a and Table S1).

Larvae and newborn adults were also adversely affected by the self library diet, as shown by a broad set of aberrant phenotypes. A small but significant fraction of F_1_ larvae displayed strong developmental delay or arrest. A fraction of newborn adult worms, in turn, displayed defective body morphology and movement (Figure 1). These defects mainly concerned body size (small animals) and gross vulval abnormalities (Figure 1b,c and Table S3). Total aberrant phenotypes affected 6.5% of viable progeny compared to less than 1% of the F_1_ offspring generated by individuals fed on OP50 as well as on non-self or self[A−] libraries.

We considered the possibility that spurious transcription of *C. elegans* fosmid clones in *E. coli*, activating some sort of unscheduled RNA interference in the developing worms, could be at the origin of the observed self-library-specific phenotypes [23]. To rule out this possibility, we analysed a mutant in RDE-1, an Argonaute protein that acts specifically on siRNAs generated in response to exogenous dsRNA [24]. Growth and development of *rde-1* mutants were indistinguishable from wild type; both genotypes showed an increase of detrimental phenotypes in the offspring of worms fed self, compared to non-self (Supplementary Table S4). This experiment excludes the involvement of the exogenous RNAi pathway due to spurious transcription from *C. elegans* fosmid cloned DNA.

We also considered the possibility that stabilisation of the ingested DNA could exacerbate the effects of the self DNA diet. To test this hypothesis, we used a mutant in the gene *nuc-1* coding for the DNaseII activity required to digest bacterial DNA in the intestine [25,26,27]. The *nuc-1* mutant strain was negatively affected by the self DNA diet, but none of the analysed phenotypes were significantly increased compared to WT (Table S5). Overall, we can conclude that recombinant *E. coli* bacteria bearing a genomic *C. elegans* library can exert negative effects on the worm when delivered by feeding. These effects can be unambiguously ascribed to the *C. elegans* DNA content of the ingested bacterial library since they are not observed when the bacteria carry heterologous DNA from a plant species in the same fosmid backbone. In addition, the induced defects strongly depend on the amount of self-DNA present in the bacteria used as a food source, self vs. self[A−], but are not increased in a mutant lacking the DNaseII enzyme acting in the intestine (Figure 1).

### 3.2. Detrimental Effects of the Self-DNA Diet across Generations

Having observed detrimental effects in the progeny of worms shifted from the standard *C. elegans* diet to the self library diet, we decided to test the performance in the course of generations, keeping worms continuously on this diet.

We selected F_1_ worms that were apparently wild type by visual inspection and individually scored them for the ability to lay eggs and give rise to healthy adults. This procedure was iterated for two additional generations.

**Figure 2:**
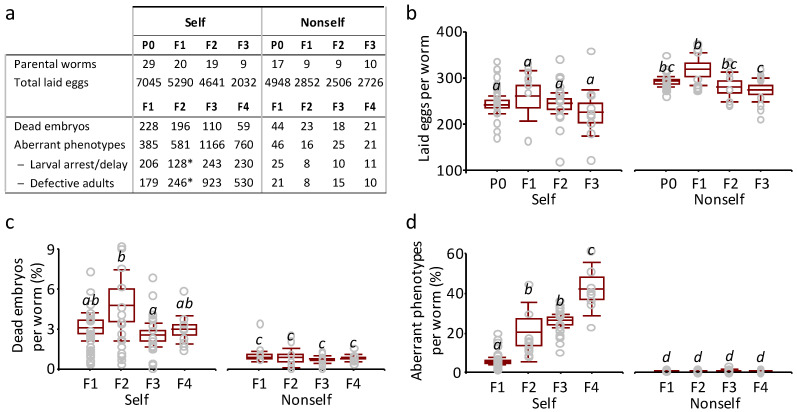
Detrimental effects of the self library diet across generations. Individual young adults of each generation were transferred to new NGM plates seeded with the bacteria genomic libraries. The total number of laid eggs, dead embryos and aberrant phenotypes per worm of each generation were scored as in Figure 1. (**a**) Screening F_1_–F_4_ progenies of worms fed self and non-self libraries. * The distinction between larval arrest/delay and defective adults in F_2_ corresponds to 374 of the total aberrant phenotypes. (**b**) Laid eggs per parental worm of each generation (P0, F1, F2 and F3). The *y*-axis reports the number of laid eggs per worm, and the *x*-axis indicates the four subsequent generations fed self (left) and non-self (right). (**c**) Dead embryos per worm (%) in F_1_–F_4_ generations. The *y*-axis reports the percentage of dead embryos per worm, and the *x*-axis indicates the four subsequent generations fed self (left) and non-self (right). (**d**) Aberrant phenotypes per worm (%) in F_1_–F_4_ generations. The *y*-axis reports the percentage of aberrant phenotypes per worm, and the *x*-axis indicates the four subsequent generations fed self (left) and non-self (right). Grey circles in (**b**–**d**) show the events per animal. Data in (**b**–**d**) refer to means, standard errors (boxes) and 95% confidence intervals (whiskers) of the dependent variables calculated for all the worms within each combination of feeding treatment and generation. For each dependent variable, different letters above bars indicate significant differences among treatments (interactive effect of feeding treatment and generation, *p* < 0.05, post hoc Tukey’s test after two-way ANOVA, details in Supplementary Tables S6 and S7).

At each generation, brood size, embryonic lethality and aberrant phenotypes were analysed in progenies of single worms fed self and non-self libraries (Figure 2). Self library feeding affected fecundity, as indicated by the small but significant reduction of brood size and embryonic viability observed in all tested generations (Figure 2a–c). In addition, newborn larvae and adults were affected as indicated by a significant increase in larval/adult aberrant phenotypes compared to the control non-self library in all generations (Figure 2a,d, Tables S6 and S7). Therefore, the detrimental effects observed in the previous experiment (Figure 1) are maintained in the course of generations.

Brood size and embryonic lethality did not change across generations (Figure 2b,c), while total aberrant phenotypes increased from one generation to another (Figure 2d, Tables S6 and S7). The brood of each worm analysed in these screenings included an increasing number (across the analysed generations) of adult worms showing different defective phenotypes but a similar fraction of larvae that did not complete their development into adults 96 h after eggs laying (Figure 2a and Figure S2).

**Figure 3:**
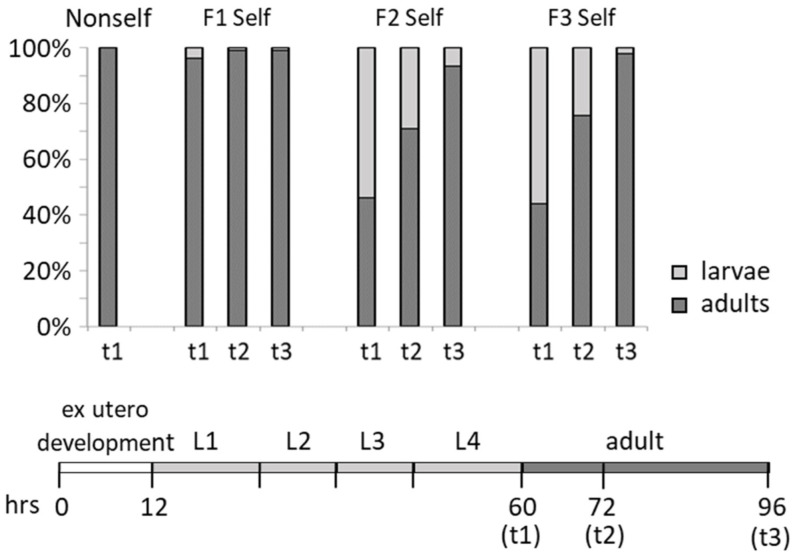
Developmental delay of F_2_ and F_3_ worms fed on the self library. Quantification of the developmental delay phenotype of F_2_ and F_3_ larvae fed self compared to larvae fed non-self. Adults were counted at each time point (hours after egg-laying, *x*-axis), and percentages of adult worms (black) and larvae (grey) were calculated. Hours required to reach each stage in wt worms fed on non-self-bacteria library and hours corresponding to t1, t2 and t3 time points are illustrated on the bar below the graph. Number of worms scored: 307 Non-self; 234 F_1_ Self; 625 F_2_ Self; 549 F_3_ Self.

In the course of this experiment, we observed that the onset of egg-laying in the worms fed on the self library was delayed compared to those fed on the control non-self library, implying that maturation to adulthood is delayed in these worms. In our standard *C. elegans* rearing conditions, post-embryonic development and larval growth, consisting of progression through four larval stages before adulthood, takes about 48 h. To get insight concerning the observed time shift across generations, timing of development, from hatching to adulthood, was analysed in worms fed self and non-self library. To this purpose, we let worms lay eggs for twelve hours and then counted the number of developed adults at regular intervals of time for a total of 96 h. All the eggs laid by worms fed on the non-self library of each generation developed into adults 60 h after deposition (Figure 3). Although more than 90% of the eggs laid by P0 worms fed with a self library were adults during the same time period, those that had not reached adulthood by this time point were still larvae after 36 more hours (96 h from deposition and counted as arrest/delay in Figure 1 and Figure 2).

A different picture emerged analysing eggs laid by F_1_ and F_2_ worms fed on the self library. About 50% of the eggs laid by these worms required a further 36 h to develop into adults (Figure 3).

In conclusion, a diet based on the self library causes embryo lethality, developmental delays (from egg hatching to adulthood) and the appearance of phenotypically aberrant worms. In particular, the progenies of F_1_ and F_2_ nematodes fed on self library displayed a delay to reach the adult stage accompanied by an increase of phenotypically altered adults; these observations suggest a negative effect on worm’s growth over the course of generations.

### 3.3. Self-DNA Feeding Induces DNA Damage and Apoptosis in the Germline

**Figure 4:**
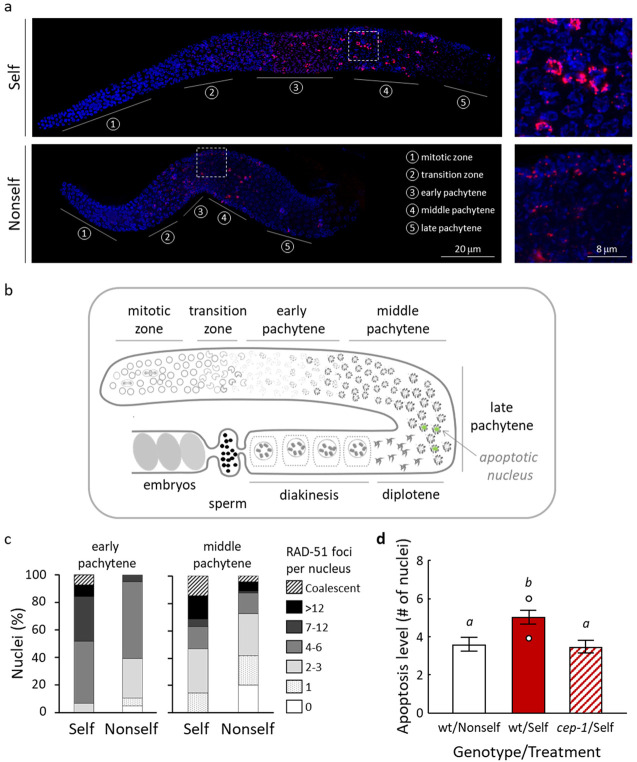
Increase of RAD-51 focus levels in early and middle pachytene nuclei and germ cell apoptosis in worms fed on the self library. (**a**) Left: representative image of dissected gonads from hermaphrodite worms fed self and non-self library. Nuclei were co-stained with anti-RAD-51 antibody (red) and DAPI (blue). Region 1 consists of mitotic nuclei, region 2 consists of meiotic nuclei in transition zone (leptotene and zygotene). Regions 3, 4 and 5 correspond to early, middle and late pachytene respectively. Scale bar, 20 μm. Right: Insets show section of main image, pachytene nuclei, indicated by white box. Scale bar, 8 μm. (**b**) Schematic representation of the *C. elegans* gonad arm. Proliferative syncytial germ cells in the mitotic zone proliferate and enter meiosis at the transition zone. Nuclei progress towards the spermatheca through the different meiotic stages. Germ cell apoptosis, green nuclei, is detected in late pachytene. (**c**) Quantification of RAD-51 foci in early and middle pachytene nuclei in worms fed self and non-self libraries. The *y*-axis represents the percentage of nuclei with the indicated number of foci. (**d**) Quantification of germline apoptosis in wt and *cep-1* worms fed indicated libraries. The *y*-axis shows the average number of SYTO-12-labelled nuclei per gonadal arm. Data refer to mean and 95% confidence interval calculated for all the gonads observed for each combination of worm genotype and library diet (a total of 90 gonadal arms were observed in three different experiments). Lettering above bars indicates significant between-group differences (*p* < 0.05, post hoc Tukey’s test after one-way ANOVA, details in Supplementary Tables S8 and S9). Images were processed using LAS X (version: 3.0.0.15697) and ImageJ (https://imagej.nih.gov/ij/ accessed on 10 October 2021) software.

Based on the above observations, we turned our attention to the hermaphroditic worm germline. In adult *C. elegans* worms, cellular proliferation only occurs in the germline, which comprises more than half of the entire set of cells of the whole body. The adult hermaphrodite worm can be thought of as a bag of cells devoted to meiosis, which are contained in two U-shaped gonadal arms joined at their proximal ends to a common uterus, where embryos, generated by fertilisation of mature oocytes, accumulate (Figure 4a,b).

Local and global changes in chromosome structure take place during germ cell development, allowing identification of different regions along the gonad (Figure 4a,b). In the “mitotic” zone, located at the distal end of each gonadal arm, reside proliferating germ cells, which enter meiosis in the following region, the “transition zone”, containing the leptotene/zygotene stages of meiotic prophase I. Meiotic DNA recombination is initiated in this region upon the formation of DSBs by the conserved topoisomerase subunit SPO-11 [28]. These DSBs are resected to generate 3′ ssDNA overhangs that are recruited by RAD-51, the eukaryotic RecA homologue, which catalyses the strand-invasion step to mediate repair by interhomolog recombination [29]. The “transition zone” is followed by the “pachytene” region, where the synaptonemal complex is completely formed and homologous chromosomes are aligned [30,31]. At the end of this meiotic stage, several germ cells undergo physiological apoptosis, while others progress into diplotene and diakinesis stages, eventually differentiating into oocytes [32].

We investigated whether meiotic progression might be altered in worms exposed to the self library. To address this question, we first examined meiotic DNA recombination by quantifying RAD-51 foci in whole-mount gonads dissected from F_1_ worms fed either the self or the non-self library. As shown in Figure 4a, where DAPI-stained chromosomes are visualised in blue and RAD-51 in red, in control non-self treated worms, RAD-51 meiotic foci emerged in the “transition zone” then accumulated in early pachytene and finally reduced in number during the mid-pachytene stage, according to the well-defined program of meiotic DSB formation and repair.

In contrast, in worms fed on self library, RAD-51 foci displayed an increase during the early and middle pachytene stages (Figure 4a). A quantitative analysis of these differences is reported in Figure 4c.

In regular conditions, the total amount and timing of meiotic DSBs occurring in *C. elegans* gonads are tightly regulated. Therefore, any alteration appearing in the spatial distribution of RAD-51 foci, as well as any sign of RAD51 foci increase and/or persistence, are indicative of deregulation of DSBs formation and/or DNA repair defects during the execution of meiosis [33,34]. In addition, unrepaired DSBs may also accumulate as a consequence of exposure to stress agents or pathogens. Whatever their origin, unrepaired DSBs are known to trigger DNA damage-dependent germ cell apoptosis mediated by p53/CEP-1 protein [35]. Moreover, we observed some nuclei in the region corresponding to the middle pachytene stage containing coalescent RAD-51 foci (Figure 4c); based on the position in which they are observed, these could be nuclei of germ cells undergoing apoptosis. Therefore, we stained with SYTO-12 germline nuclei undergoing apoptosis and scored them in F_1_ worms fed with either library. Worms fed on the non-self library exhibited less than four apoptotic bodies in late pachytene, as expected for physiological apoptosis (Figure 4d). A different picture emerged in the gonads of worms fed on the self library, which displayed a significant increase of apoptotic levels. Notably, increased germ cell apoptosis was p53/CEP-1 dependent (Figure 4d, Supplementary Tables S8 and S9), suggesting that activation of a DNA damage checkpoint occurs in the gonads of worms fed on self library. Having observed an increase of negative effects across generations, we scored apoptotic nuclei also in gonads of F_2_ hermaphrodites. We found that F_2_ worms exhibited an apoptotic level similar to F_1_ worms (F_1_ Self = 4.88 ± 0.08, F_1_ Non-self = 3.55 ± 0.2, F_2_ Self = 4.99 ± 0.36, F_2_ Non-self =3.22 ± 0.35), suggesting that damaged nuclei detected by the meiotic checkpoint do not increase across generations and in agreement with the observation that laid eggs and embryo lethality do not change across generations (Figure 2).

Meiotic progression is temporally and spatially regulated; nuclei at any given position along the gonadal arm have spent the same time in meiosis and rows of nuclei share cytological landmarks specific to different meiotic stages (above, Figure 4). In the “transition zone”, the chromatin is concentrated on one side, and nuclei appear as crescent-shaped by DAPI staining; in pachytene, chromatin appears redistributed throughout the entire nuclear periphery; in diplotene (corresponding to the gonad bend or loop region) homologs lose their associations, the DAPI signal becomes diffuse throughout the entire nucleus and chromatin continues to condense in preparation for diakinesis [36].

**Figure 5:**
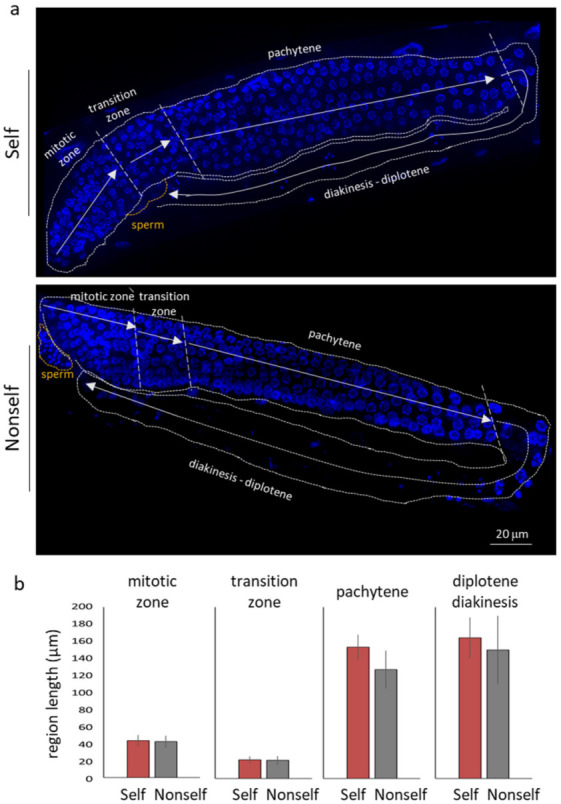
Regular progression of nuclear stages along the gonadal arms of worms fed on the self library. (**a**) Images of DAPI-stained germline in hermaphrodite worms fed self and non-self library. The dashed white lines surround the germline syncytium. The different regions of the gonad are demarcated by vertical dashed white lines and indicated with arrows. The dashed orange lines surround the sperm nuclei. Scale bars represent 20 µm. (**b**) Quantification of length of different regions in hermaphrodite germline fed indicated libraries. The *y*-axis shows the mean length of regions (µm). Error bars correspond to SD. Nine germlines were analysed per diet. Images were processed using LAS X (version: 3.0.0.15697) and ImageJ (https://imagej.nih.gov/ij/, accessed on 10 October 2021) software.

In order to compare meiotic progression in nematodes fed self and non-self bacteria, we visualised DAPI stained nuclei in whole mounted animals (Figure 5). The lengths of the different stages along the arms were used as a proxy to measure the progression (or stalling) of the different stages, and the resulting averaged lengths were compared (Figure 5b). This cytological analysis did not reveal significant differences introduced by the self-bacterial diet.

**Figure 6:**
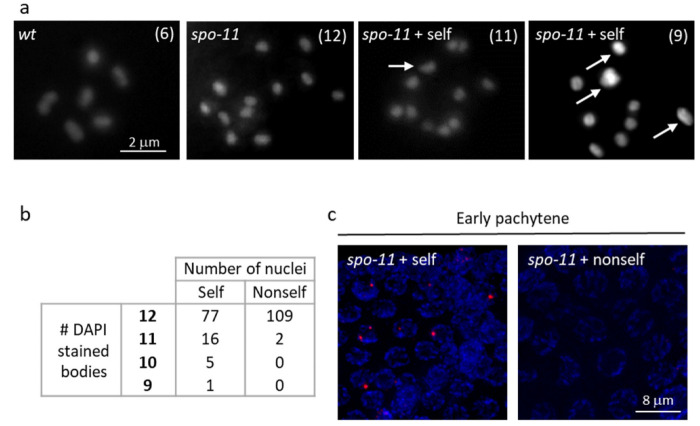
DNA damage occurs via an SPO-11-independent pathway in gonads of worms fed on the self library. (**a**) Representative images of wt and *spo-11* DAPI-stained oocyte nuclei at diakinesis. The number in parenthesis within each image indicates the number of DAPI-stained bodies that were detectable through the z stack of the nucleus. The arrows indicate the bivalents in *spo-11* worm fed on the self library (*spo-11* + self). Scale bar, 2 μm. (**b**) Quantification of DAPI stained body in diakinesis nuclei in *spo-11* mutants fed on the two libraries. (**c**) Representative images of early pachytene nuclei in gonads of *spo-11* worms, fed on self and non-self libraries, co-stained with anti-RAD-51antibody (red) and DAPI (blue). Scale bars, 8 μm. Images were processed using LAS X (version: 3.0.0.15697) and ImageJ (https://imagej.nih.gov/ij/, accessed on 10 October 2021) software.

Lack of evidence of meiotic progression alteration in the presence of activation of the DNA damage checkpoint (above) prompted us to analyse the impact of the self library in *spo-11* mutant worms. In the absence of SPO-11, meiotic DSBs are not formed; consequently, RAD-51 is not recruited to chromosomes, and diakinesis nuclei show 12 DAPI-stained bodies corresponding to twelve univalents, in place of six bivalents consisting of homolog chromosomes held together by chiasmata [28,29] (Figure 6a). Strikingly, in *spo-11* mutant worms fed on the self library, approximatively 16% of oocyte nuclei showed eleven DAPI-stained bodies, corresponding to ten univalents and one bivalent, and 5% of oocyte nuclei showed ten DAPI-stained bodies, corresponding to eight univalents and two bivalents (Figure 6a,b). These data indicate the occurrence of SPO-11 independent DNA ruptures in worms fed on the self library, as further confirmed by the presence of RAD-51 foci in *spo-11* germline nuclei during the early pachytene stage (Figure 6c). In conclusion, germline nuclei of worms fed on the self library show DNA damage that is SPO-11 independent and an increase of apoptosis that is p53/CEP-1 dependent.

## 4. Discussion

Evidence that fragmented extracellular DNA (exDNA) produces a concentration-dependent, species-specific inhibitory effect has been reported in plants and other organisms, including microbes, fungi, protozoa and insects [3,4,6].

In plants, following self- and non-self-DNA recognition at the root level, different cascades of events trigger specific molecular responses, with self-DNA inducing membrane depolarisation [37] and ROS accumulation [6,38].

Here, we investigated the effects of self-DNA exposure in *C. elegans*. This organism has been widely used as an infection model to study how it responds to and how it recognises PAMPs [39,40], but only very recently, it was explored as a model to study the effects of non-self cytoplasmic DNA [41]. In our experiments, the effects of self-DNA exposure, mediated by a diet based on bacteria bearing a *C. elegans* genomic library, were compared to the effects of a control diet based on bacteria bearing an *M. truncatula* genomic library. Both fosmid libraries were characterised by large genomic inserts and the inducibility of fosmid copy number. We found that a self library diet specifically affects developmental and reproductive processes and results in DNA damage in the *C. elegans* germline.

Adult P0 worms fed on self library were apparently as healthy as those fed on non-self library except for a decrease in brood size (Figure 2b). Progenies of all analysed generations showed increased embryo lethality (Figure 2c). A delay of development, from embryo to adulthood, was observed in F_1_ and F_2_ progenies (Figure 3). A fraction of adult worms appeared defective, and the fraction of defective adults increased over the analysed generations (Figure S2). The progeny of each worm fed on self library showed a number of different aberrant phenotypes. These phenotypes may arise from somatic alteration occurring during embryonic or larval development, but we cannot exclude that they may arise from genetic alteration due to induced genomic instability. In adult worms, only germ cells devoted to gamete production proliferate and differentiate as they move along the gonad. Progression of gametes development during meiosis can be affected by a number of factors that include food, developmental rate and genotoxic stresses. Our cytological analysis revealed subtle but specific effects on meiotic nuclei of F_1_ worms fed on the self library (Figure 4 and Figure 6). First, DSBs, indirectly detected by RAD-51 immunostaining, were more abundant during early and middle pachytene stages, a sign of increased and/or unrepaired DNA damage. Second, a CEP-1-dependent increase of apoptotic nuclei in the gonads suggests DNA damage detection. Third, RAD-51 immunostaining revealed foci in a *spo-11* mutant fed self library, suggesting that the excess of RAD-51 foci observed in wild type is not dependent on DSBs operated by the meiosis-specific nuclease SPO-11. RAD-51 foci, revealed on meiotic prophase chromosomes, imply active DNA damage repair. At least three observations indicate that meiotic DNA repair/recombination is not specifically affected in worms fed on the self library: (i) regular progression of meiotic stages along the gonads was observed (Figure 5); (ii) the frequency of male progeny, determined by X chromosome non-disjunction events, is not increased in wild-type worms (data not shown); (iii) *spo-11* mutant oocytes at diakinesis showed, in place of the typical twelve unpaired chromosomes (univalents) as observed in *spo-11* mutant fed non-self library, a significant number of bivalents, from one to three; this implies that DNA repair occurs and results in linked pairs of chromosomes (Figure 6). During embryogenesis, cell cycle delay to repair DNA damage is suppressed, and rapid cell division is favoured over accurate DNA replication [42,43]. DNA damage during this developmental stage could be at the origin of the observed developmental defects in the progenies of worms fed self library.

Although a clear explanation for these detrimental phenotypes cannot be provided, our experiments point to a specific effect mediated by the *C. elegans* genomic sequences present on the bacterial fosmid library. We excluded that the formation of *C. elegans* specific small RNA in the fosmid-carrying bacteria host could be at the origin of the phenotypes. Moreover, observed detrimental effects are sharply dependent on fosmid copy number (Figure 1), and this excludes altogether potential mutations in any *E. coli* host clones. Such mutations could arise unnoticed in the complexity of the entire library but may result in toxicity to *C. elegans*. We cannot exclude that specific fosmid clones are involved in the adverse response, although feeding worms with a subset of a few randomly chosen clones led to effects comparable to those observed when feeding them on the entire library (Figure 1).

Cytoplasmic DNA-sensors have not been discovered in *C. elegans*, but it has been recently demonstrated that persistent, *E. coli* derived, cytoplasmic DNA leads to detrimental effects and loss of functionality in the analysed tissues [41]. These authors found that all the observed tissue degeneration phenotypes were fully alleviated by loss of FSHR-1, a G protein-coupled receptor required for the activation of infection and stress response genes [44]. Adverse effects of cytoplasmic foreign DNA were manifested in a *nuc-1* mutant defective in DNase II activity, that in *C. elegans* is required to digest the DNA of dead cells during engulfment and to digest bacterial DNA in the gut [25,26,27]. Finally, they revealed that lack of activation or maintenance of protein folding stress (UPR) is at the basis of the adverse effects and that therapeutic activation (drug mediated) of the ER unfolded protein response was sufficient to revert the deleterious phenotypes. DNase II activity associated with NUC-1 does not appear required to alleviate the effect of self-DNA exposure in the *C. elegans* diet (Table S5). This result was unexpected and might be attributed to the elevated expression of antimicrobial genes in *nuc-1* mutants [45] that could have a beneficial cytoprotective effect in the tested conditions.

The observed detrimental effects in wild-type worms fed on self library include DNA damage in meiotic nuclei at molecular level. DNA damage repair has been linked to innate immune programmes in mammals [46], and “germline DNA damage-induced stress resistance” (GDISR) is a well-documented example that has been provided in *C. elegans* [10]. These authors found that GDISR is triggered by MPK-1, a MAP kinase protein involved in many processes during germline development, including apoptosis, and in the expression of immune genes during *C. elegans* infections. GDISR not only results in systemic stress resistance but also promotes defence against bacterial infections in the intestine [10,47].

In this work, we observed the formation of SPO-11 independent DSBs in *C. elegans* germline nuclei of worms fed on a self-DNA library. Very recently, SPO-11 independent DSBs have been linked to the mobilisation of transposable elements [48,49]. Additionally, activation of specific transposons has been demonstrated in worms exposed to environmental stresses [49,50,51]. This raises the intriguing possibility that transposon activation might be one of the outcomes of self-DNA exposure. Transposon mobilisation is under the control of specific regulatory pathways. Among these, small RNAs that induce RNA degradation and/or deposition of repressive epigenetic modifications and DNA chemical modifications have been reported [52]. It is worth noting that changes in 5-methylcytosine levels have been associated with self-DNA exposure in *plants* [38]. This DNA modification appears to be absent in *C. elegans* but the presence of adenine N^6^-methylation has been documented [53] and might be associated with transposition regulation [54]. These considerations indicate a field of further investigation to elucidate the specific pathways activated by a self-DNA enriched diet in *C. elegans*.

In general, we cannot yet assess the direct or indirect effect of ingested self-DNA on DNA damage. For example, DNA damage might result from an overproduction of reactive oxygen species (ROS), as seen, for example, during intestinal infections [55]. Interestingly, an increase in ROS formation was among the dose-dependent and species-specific immunity-related traits induced in common bean treated with fragmented self-DNA [5].

Summarising our results, we demonstrated that an inhibitor effect of self-DNA also occurs in the model nematode *C. elegans*. We also found that this effect can be obtained in worms by feeding them with bacterial cells carrying a *C. elegans* genomic library. Adverse effects were observed in the progenies of worms exposed to self-DNA via their bacterial diet.

## 5. Conclusions

This work sets the stage for the investigation of the molecular mechanisms underlying the inhibitory effect of extracellular self-DNA in *C. elegans*.

Whatever the mechanism involved, it will be interesting to explore potential applications of the observed phenomenon for biocontrol in both agriculture and animal and human medicine [56]. In fact, the use of a microbial library is a proof of concept that an upscaling of production of DNA of a target pathogenic species, and its delivery through the diet, is possible.

Environmental DNA is ubiquitous. It has been described in many habitats, including soil, sediments, oceans and freshwater [57,58,59], and it is obviously present in the diets of all animal species. Our findings confirm that a diet containing heterologous DNA has no negative effects, and thus, the use of DNA of pathogens/parasites as food integrators can be, at the same time, not harmful to the host while inhibiting a target species.

## Data Availability

The data used to support the findings of this study are included within the article and the supplemental file.

## Supplementary Materials

The following are available online at https://www.mdpi.com/article/10.3390/biology11020262/s1, Figure S1: Fosmid copy number increased upon addition of L-arabinose. Figure S2. Separation of larval arrests/delays and defective adults across generations. Table S1: Effects of parental feeding treatments on the occurrence of embryonic lethality and aberrant phenotypes in the progeny of *C. elegans* (see Figure 1a–b in main text). Table S2. List of fosmids of the *C. elegans* library (self subset) used in Figure 1. Table S3. Phenotypes of defective adults. Table S4. Effects on progenies of wild type and *rde-1* mutant worms fed on self or nonself libraries. Table S5. Effects on progenies of WT and *nuc-1* mutant worms fed on self or non-self libraries. Table S6. Results of two-way ANOVA testing for the effects of feeding treatment (either Self or Nonself), generation (four levels) and their interaction on the number of laid eggs per worm and the per cent frequency of dead embryos and aberrant phenotypes in the progeny of *C. elegans* (see Figure 2b–d in main text). Table S7. Effects of parental feeding treatment with either Self or Nonself libraries on the number of laid eggs and per cent frequency of dead embryos and aberrant phenotypes in the progeny of *C. elegans* across four different generations (see Figure 2b–d in main text). Table S8. Results of one-way ANOVA testing for significant diet and worm genotype differences on the average number of SYTO-12-labelled nuclei per gonadal arm (see Figure 4d in main text). Table S9. Effects of diet and worm genotype differences on the average number of SYTO-12-labeled nuclei per gonadal arm (see Figure 4d in main text). For each combination of diet and worm genotype, data refer to mean and 95% confidence interval calculated on a randomized, replicated sample (N ≥ 90). Results of statistical analyses refer to significant pairwise differences, showed as P values resulting from Tukey’s post hoc HSD test for unequal sample sizes, after one-way ANOVA shown in Supplementary Table S8.
