# Supplementary material for: Self-DNA Exposure Induces Developmental Defects and Germline DNA Damage Response in Caenorhabditis elegans
Source: Biology (Basel). 2022 Feb 8;11(2):262. doi: 10.3390/biology11020262 (PMC8869574; doi:10.3390/biology11020262)
Supplement: Supplementary file 1 [file biology-11-00262-s001.zip › biology-1577233-supplementary.pdf]

Self-DNA Exposure Induces Developmental Defects and Germline DNA Damage Response in *Caenorhabditis elegans*

Marcello Germoglio, Adele Adamo, Guido Incerti, Fabrizio Cartenì, Silvia Gigliotti, Aurora Storlazzi and Stefano Mazzoleni

Supplementary Materials:

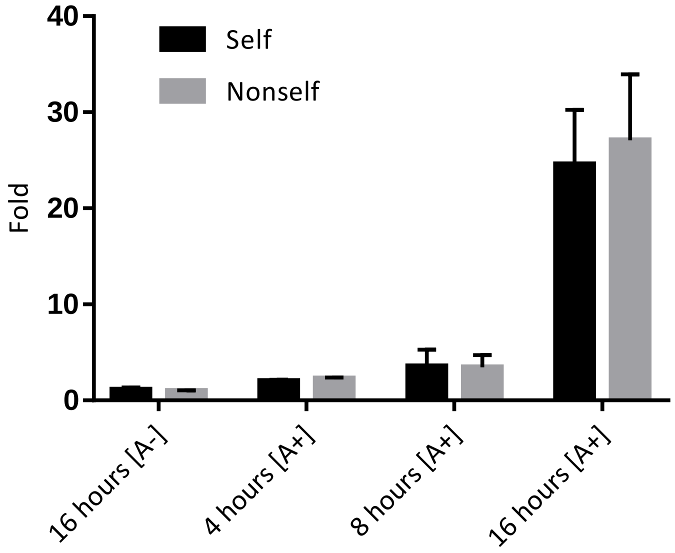

Scheme S1. Fosmid copy number increased upon addition of L-arabinose. The graph shows the fold change (relative copy number of fosmids) in *C. elegans* and *Medicago truncatula* libraries grown 4hrs, 8hrs, 16 hrs in LB supplemented with L-arabinose [A+]. Bars indicate standard deviation from 3 technical replicates.

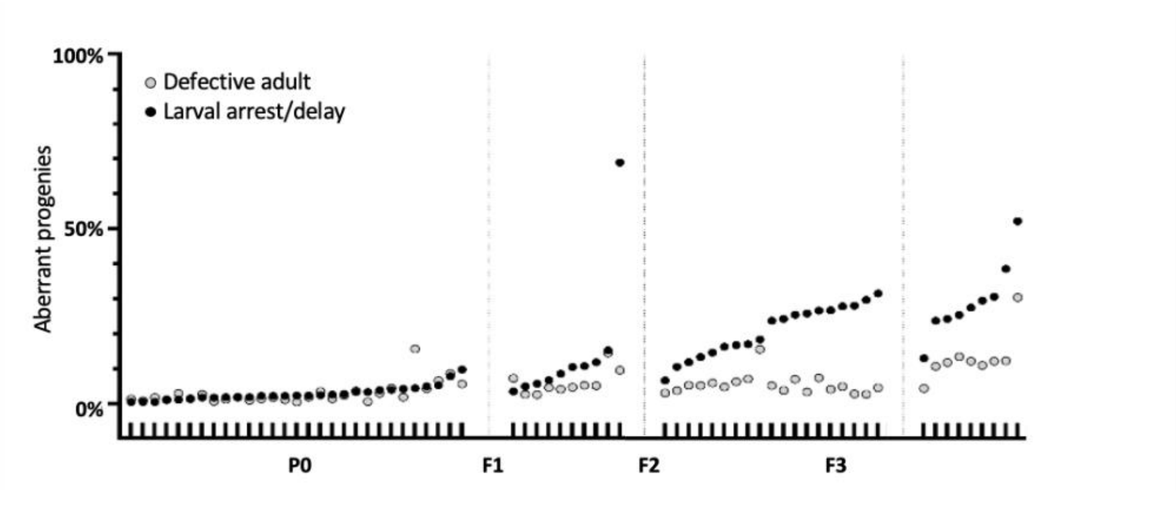

Scheme S2. Separation of larval arrests/delays and defective adults across generations. The x-axis indicates parental worms in each generation (comb teeth) fed on self library. The y-axis reports the percentages of defective adults (black) and larval arrests/delays (grey) per worm.

Supplementary Table S1. Effects of parental feeding treatments on the occurrence of embryonic lethality and aberrant phenotypes in the progeny of *C. elegans* (see Fig. 1a-b in main text). For each treatment and effect, data refer to median and interquartile range of percent frequency of the effect, calculated on the F_1 progeny of a variable number (N) of randomized, replicated worms. Results of statistical analyses testing for overall treatment effect, as well as for pairwise effect comparisons between Self and either other treatments are also shown, as P values resulting from Kruskall-Wallis ANOVA and Mann-Whitney U tests, respectively.

Effects	Treatment						Statistical analysis		
	Self	N	Self [A-]	N	Nonself	N	P (K-W ANOVA)	P (Self = Self [A-])	P (Self = Nonself)
Embryonic lethality (%)	2.64 (2.15 ÷ 4.31)	15	0.51 (0.32 ÷ 0.63)	10	0.70 (0.64 ÷ 1.08)	10	< 0.00001	0.00016	0.00059
Aberrant phenotypes (%)	3.86 (2.63 ÷ 9.09)	15	0.38 0.32 ÷ 0.65)	10	0.96 (0.72 ÷ 1.04)	10	< 0.00001	0.00032	0.00040

Effects	Treatment				Statistical analysis	
	OP50	N	Self-subset	N	P (Self = Self-subset)	P (Nonself = OP50)
Embryonic lethality (%)	1.02 (0.61 ÷ 1.20)	8	2.30 (1.61 ÷ 3.54)	16	0.34278	0.53396
Aberrant phenotypes (%)	0.20 (0.00 ÷ 0.80)	8	3.48 (2.52 ÷ 4.86)	16	0.46456	0.25198

Supplementary Table S2. List of fosmids of the C. elegans library (self subset) used in Figure 1

Chromosome	Clone	Clone size	Clone start	Clone end
I	WRM0610bA07	31172	7230131	7261303
I	WRM0610aA11	34068	7974014	8008082
II	WRM0610aA05	34429	4905085	4939514
II	WRM0610aA04	19714	8289863	8309577
II	WRM0610bA11	33968	12465047	12499015
III	WRM0610bA10	27446	137548	164994

III	WRM0610aA09	32802	6044368	6077170
III	WRM0610aA10	34196	6488679	6522875
IV	WRM0610bA01	35823	1511885	1547708
IV	WRM0610dA01	34487	4234121	4268608
IV	WRM0610bA06	37647	11539101	11576748
V	WRM0610aA08	23116	5071293	5094409
V	WRM0610aA06	30838	7113128	7143966
X	WRM0610bA04	35806	1144437	1180243
X	WRM0610aA12	30483	8624052	8654535
X	WRM0610cA02	33998	12671842	12705840
X	WRM0610aA03	29543	12732166	12761709

Supplementary Table S3. Phenotypes of defective adults.

Abnormal body length and shape: Sma and Lon (worms that have shorter or longer body lengths compared to wt); Dpy (worms short and fat, dumpy).

Abnormal vulva / gonad: Vul: worms that showed an abnormal development of the vulva (vulva-less or protruding vulva); Muv (the worms have two or more vulva, multi-vulva); Gon (worms with abnormal gonad development. The gonad appears with little or no tubular morphology).

Uncoordinated movement: Unc (worms that fail to correctly move, that display poor forward movement or that fail to move backwards).

	OP50	Nonself	Self	Self [A-]	Self subset
Hatched progeny	2621	2862	3712	3084	4073
No. Larval arrest/delay (%)	5 (0.19)	10 (0.35)	138 (3.72)	7 (0.23)	116 (2.85)
No. Defective adults (%)	2 (0.08)	16 (0.56)	111 (2.99)	8 (0.26)	51 (1.25)
Phenotypes of defective adults:					
Abnormal body length and shape	1	13	72	7	26
Abnormal vulva or gonad	0	3	26	1	14
Uncoordinated movement	1	0	13	0	11

Supplementary Table S4. Effects on progenies of wild type and *rde-1* mutant worms fed on self or nonself libraries.

Different letters indicate significant differences among combinations of genotype and diet within each dependent variable (table row), according to pairwise testing with Mann-Whitney U test at $P < 0.05$, on data expressed per worm.

**Genotype**	**wt**	**wt**	***rde-1***	***rde-1***
**Diet**	**Self**	**Nonself**	**Self**	**Nonself**
Parental worms	8	6	8	6
Laid eggs	1938	1501	1615	1609
Dead embryos	35 [b]	11 [a]	29 [b]	12 [a]
Aberrant phenotypes	73 [b]	27 [a]	112 [c]	45 [a]
- Larval arrest/delay	27	12	37	16
- Defective adults	46	15	75	29

Supplementary Table S5. Effects on progenies of WT and *nuc-1* mutant worms fed on self or nonself libraries. Different letters indicate significant differences among combinations of genotype and diet within each dependent variable (table row), according to pairwise testing with Mann-Whitney U test at P < 0.05, on data expressed per worm.

**Genotype**	**wt**	***nuc-1***	***nuc-1***	**wt**	***nuc-1***
**DIET**	**Self**	**Self**	**Nonself**	**Self subset**	**Self subset**
Parental worms	8	10	8	8	8
Laid eggs	1909	2691	2115	2096	1944
Dead embryos (%)	42 ^{b} (2.20)	82 ^{b} (3.05)	14 ^{a} (0.66)	67 ^{b} (3.20)	50 ^{b} (2.57)
Aberrant phenotypes (%)	90 ^{d} (4.82)	153 ^{d} (5.86)	9 ^{c} (0.43)	98 ^{d} (4.83)	92 ^{d} (4.86)
-Larval arrest/delay (%)	39 (2.09)	92 (3.53)	5 (0.24)	47 (2.32)	47 (2.48)
- Defective adults (%)	51 (2.73)	61 (2.34)	4 (0.19)	51 (2.51)	45 (2.38)

Supplementary Table S6. Results of two-ways ANOVA testing for the effects of feeding treatment (either Self or Nonself), generation (four levels) and their interaction on the number of laid eggs per worm and the percent frequency of dead embryos and aberrant phenotypes in the progeny of *C. elegans* (see Fig. 2b-d in main text).

**Effect**	**df**	**SS**	**MS**	**F**	**P**
Laid eggs per worm (n)					
Treatment (T)	1	52361	52361	22.27	<0.0001
Generation (G)	3	20614	6871	2.92	0.0370
T x G	3	1157	386	0.16	0.9204
Error	114	268039	2351		
Dead embryos (%)					
Treatment (T)	1	163.80	163.80	19.30	< 0.0001
Generation (G)	3	19.64	6.55	0.77	0.5124
T x G	3	16.43	5.48	0.65	0.5875
Error	114	967.49	8.49		
Total aberrant phenotypes (%)					
Treatment (T)	1	11608.39	11608.39	147.56	< 0.0001
Generation (G)	3	4940.93	1646.98	20.94	< 0.0001
T x G	3	4964.64	1654.88	21.04	< 0.0001
Error	114	8968.02	78.67		

Supplementary Table S7. **Effects of parental feeding treatment with either Self or Nonself libraries on the number of laid eggs and percent frequency of dead embryos and aberrant phenotypes in the progeny of *C. elegans* across four different generations (see Fig. 2b-d in main text).** For each effect, treatment and generation, data refer to mean and 95% confidence interval calculated on a randomized, replicated sample (N ranging between = 9 to 29). Results of statistical analyses refer to significant pairwise differences between treatments or between generations, showed as P values resulting from Tukey's post-hoc HSD test for unequal sample sizes, after two-ways ANOVA shown in Table S6.

Effects	Treatment	Generation				Statistical analysis				
		**P0**	**F1**	**F2**	**F3**	**P**<br>**P0 vs. F1**	**P**<br>**F1 vs. F2**	**P**<br>**F2 vs. F3**	**P**<br>**P0 vs. F3**	**P**<br>**Self vs. Nonself**
Laid eggs per worm (n)	Self	243<br>(224 ÷ 262)	264<br>(235 ÷ 294)	244<br>(221 ÷ 267)	226<br>(176 ÷ 276)	0.8525	0.9022	0.9924	0.9952	*0.0002*
	Nonself	291<br>(280 ÷ 302)	317<br>(282 ÷ 352)	278.4<br>(248 ÷ 309)	272.6<br>(246 ÷ 299)	0.9489	0.6989	0.9999	0.9896	
		**F1**	**F2**	**F3**	**F4**	**P**<br>**F1 vs. F2**	**P**<br>**F2 vs. F3**	**P**<br>**F3 vs. F4**	**P**<br>**F1 vs. F4**	**P**<br>**Self vs. Nonself**
Dead embryos (%)	Self	3.1<br>(2.1 ÷ 4.2)	4.8<br>(2.0 ÷ 7.5)	2.5<br>(1.6 ÷ 3.4)	3.0<br>(1.9 ÷ 4.0)	0.6447	0.2645	0.9999	0.9999	*0.0002*
	Nonself	0.9<br>(0.5 ÷ 1.3)	0.9<br>(0.1 ÷ 1.6)	0.7<br>(0.4 ÷ 1.0)	0.8<br>(0.5 ÷ 1.1)	0.9999	0.9999	0.9999	0.9999	
Aberrant phenotypes (%)	Self	5.7<br>(3.9 ÷ 7.5)	14.3<br>(6.9 ÷ 21.8)	26.2<br>(22.5 ÷ 29.8)	42.5<br>(29 ÷ 55.9)	0.0506	*0.0019*	*0.0040*	*0.0001*	*0.0001*
	Nonself	0.9<br>(0.8 ÷ 1.0)	0.5<br>(0.2 ÷ 0.9)	1.0<br>(0.2 ÷ 1.8)	0.8<br>(0.3 ÷ 1.2)	0.9999	0.9999	0.9999	0.9999	

Supplementary Table S8. **Results of one-way ANOVA testing for significant diet- and worm genotype-differences on the average number of SYTO-12-labeled nuclei per gonadal arm (see Fig. 4d in main text).**

Effect	df	SS	MS	F	P
Diet/genotype	2	204.68	102.34	23.377	<0.0001
Error	357	1562.86	4.38		

Supplementary Table S9. Effects of diet- and worm genotype-differences on the average number of SYTO-12-labeled nuclei per gonadal arm (see Fig. 4d in main text). For each combination of diet and worm genotype, data refer to mean and 95% confidence interval calculated on a randomized, replicated sample (N ≥ 90). Results of statistical analyses refer to significant pairwise differences, showed as P values resulting from Tukey's post-hoc HSD test for unequal sample sizes, after one-way ANOVA shown in Supplementary Table S8.

	Diet / genotype						Statistical analysis		
**Effects**	**wt / Self**	**N**	**cep-1 / Self**	**N**	**wt / Nonself**	**N**	**P** wt/Self = *cep-1*/Self	**P** wt/Self = wt/Nonself	**P** *cep-1*/Self = wt/Nonself
N of SYTO-12-labeled nuclei per gonadal arm	5.1 (4.7 ÷ 5.4)	180	3.5 (3.2 ÷ 3.8)	90	3.6 (3.3 ÷ 3.9)	90	0.000023	0.000032	0.91885
